# Expression Analysis of the Hippo Cascade Indicates a Role in Pituitary Stem Cell Development

**DOI:** 10.3389/fphys.2016.00114

**Published:** 2016-03-31

**Authors:** Emily J. Lodge, John P. Russell, Amanda L. Patist, Philippa Francis-West, Cynthia L. Andoniadou

**Affiliations:** Craniofacial Development and Stem Cell Biology, Dental Institute, King's College LondonLondon, UK

**Keywords:** pituitary, Hippo, YAP1, TAZ, pituitary stem cells, Rathke's pouch

## Abstract

The pituitary gland is a primary endocrine organ that controls major physiological processes. Abnormal development or homeostatic disruptions can lead to human disorders such as hypopituitarism or tumors. Multiple signaling pathways, including WNT, BMP, FGF, and SHH regulate pituitary development but the role of the Hippo-YAP1/TAZ cascade is currently unknown. In multiple tissues, the Hippo kinase cascade underlies neoplasias; it influences organ size through the regulation of proliferation and apoptosis, and has roles in determining stem cell potential. We have used a sensitive mRNA *in situ* hybridization method (RNAscope) to determine the expression patterns of the Hippo pathway components during mouse pituitary development. We have also carried out immunolocalisation studies to determine when YAP1 and TAZ, the transcriptional effectors of the Hippo pathway, are active. We find that YAP1/TAZ are active in the stem/progenitor cell population throughout development and at postnatal stages, consistent with their role in promoting the stem cell state. Our results demonstrate for the first time the collective expression of major components of the Hippo pathway during normal embryonic and postnatal development of the pituitary gland.

## Introduction

The pituitary gland is a critical endocrine organ that controls multiple essential physiological processes such as metabolism, stress response, growth and reproduction. It is not surprising therefore, that abnormal pituitary function leads to human disease, including hypopituitarism and pituitary tumors, which can be associated with high morbidity and mortality. Hypopituitarism has an estimated prevalence of 45.5 per 100,000 (Schneider et al., 2007) and clinically relevant pituitary adenomas are reported to have a mean prevalence of 94 per 100,000 although up to one in six individuals are found to carry pituitary microadenomas (Ezzat et al., 2004; Daly et al., 2006). Understanding the genes and pathways that control normal pituitary development and function, and their likely involvement in disease, is required to speed up the discovery of new tools to improve patient management.

The pituitary develops from two discrete embryonic tissues; oral ectoderm, which gives rise to the endocrine anterior pituitary comprised of the intermediate and anterior lobes, and neural ectoderm (ventral diencephalon), which gives rise to the posterior pituitary, which is connected with the hypothalamus. In mice, the hypophyseal placode, the primordium of the anterior pituitary, is first identifiable as a thickening in the oral ectoderm at 8.0 days *post coitum* (dpc). From 9.0dpc the placode invaginates, forming the anterior pituitary primordium termed Rathke's pouch (RP). The overlying ventral diencephalon then evaginates toward and contacts RP by 10.5dpc to form the infundibulum (de Moraes et al., 2012; Rizzoti, 2015). Subsequently, RP detaches from the oral epithelium, to form the definitive pouch by 12.5dpc. The definitive pouch retains a central lumen that is lined by SOX2^+^ uncommitted progenitor cells (de Moraes et al., 2012; Rizzoti, 2015). Descendants of these SOX2^+^ cells restrict their fate to three lineages (Fauquier et al., 2008; Andoniadou et al., 2013; Rizzoti et al., 2013), which are characterized by expression of transcription factors PIT1, TPIT, and SF1. PIT1^+^ progenitor cells differentiate into prolactin-secreting lactotrophs, growth hormone-secreting somatotrophs and thyroid-stimulating hormone-secreting thyrotrophs; TPIT^+^ progenitors give rise to adrenocorticotrophic hormone-secreting corticotrophs in the AP and melanocyte-stimulating hormone-secreting melanotrophs in the IL. Lastly, SF1^+^ progenitors produce luteinizing hormone- and follicle-stimulating hormone-secreting gonadotrophs. A proportion of SOX2^+^ cells (3–5% of total pituitary cells) persist into adult life (Fauquier et al., 2008; Jayakody et al., 2012; Andoniadou et al., 2013). Postnatally, these SOX2^+^ cells are predominantly found in a thin epithelial layer between the anterior and intermediate lobes of the pituitary (marginal zone), and groups of SOX2^+^ cells are also dispersed within the parenchyma.

Multiple signals are required for correct pituitary development, however the activity or role of the Hippo pathway has not been previously studied. The infundibulum expresses FGF8, FGF10, and BMP2 from 9.0dpc (Treier et al., 1998, 2001), which diffuse to form a dorsal-ventral gradient. Changes in the extent of these expression domains within the infundibulum directly influence anterior pituitary size. In the absence of FGF signaling, RP is initially specified but cells fail to proliferate and undergo apoptosis (De Moerlooze et al., 2000; Ohuchi et al., 2000). FGF activity is mediated through the transcription factor LIM Homeobox 3 (LHX3) (Ericson et al., 1998), required for progenitor specification and proliferation (Sheng et al., 1997). SHH is expressed in the non-hypophyseal oral ectoderm and ventral diencephalon surrounding the infundibulum and signals to the developing RP (Treier et al., 2001; Khonsari et al., 2013). Loss of SHH signaling leads to a reduction in pituitary tissue, a phenotype attributed both to defective patterning and proliferation. The mesenchyme around the developing pituitary, derived from the neural crest rostrally and from the paraxial mesoderm caudally (Jiang et al., 2002; McBratney-Owen et al., 2008), expresses WNT and BMP signals that also influence morphogenesis, proliferation and cell-fate specification (Treier et al., 1998; Davis and Camper, 2007). WNT ligands have a role in promoting pituitary progenitor proliferation and PIT1-lineage specification as well as for correct expression of FGF and BMP factors (Cha et al., 2004; Potok et al., 2008; Gaston-Massuet et al., 2011; Andoniadou et al., 2013). Similarly, BMP2 and BMP4 are required for pituitary growth and lineage specification (Takuma et al., 1998; Treier et al., 1998).

The Hippo pathway regulates organ size through the control of stem cell activity, proliferation and apoptosis. The pathway is an inhibitory phosphorylation cascade first identified in *Drosophila*, where mutations in the Hippo (Hpo) kinase led to over-proliferation in imaginal discs and tissue overgrowth in the adult fly (Wu et al., 2003). The mammalian core Hippo pathway consists of MST1/MST2 kinases (Hpo homologs, a.k.a. STK4/STK3), which activate LATS1/LATS2 kinases, leading to phosphorylation of effectors YAP1 and TAZ (WWTR1) at multiple sites. These are then retained in the cytoplasm via 14-3-3 (Hao et al., 2008) or ubiquitinated and degraded (Zhao et al., 2011). When the Hippo pathway is not active, YAP1 and TAZ can enter the nucleus and bind to transcription factors TEAD1-4 (Ota and Sasaki, 2008; Zhao et al., 2008), to activate transcription of stemness, proliferation and anti-apoptotic genes. Loss of function of core kinases leads to increased proliferation in several tissues, most obviously in the liver (Lu et al., 2010), heart (Heallen et al., 2011, 2013), and intestine (Cai et al., 2010; Imajo et al., 2015).

YAP1/TAZ regulate proliferation, survival and differentiation and are active in many stem cell populations including embryonic stem cells (Lian et al., 2010). Many factors have been implicated in the regulation of YAP1/TAZ via modulation of the core Hippo pathway. These include the proto-cadherins FAT4 and DCHS1 (Cappello et al., 2013; Bagherie-Lachidan et al., 2015) where loss of FAT4/DCHS1 has been reported to result in an increase in YAP1 and TAZ activity in neuronal cells and nephron progenitors. SOX2 has been shown to antagonize the Hippo pathway, leading to the nuclear accumulation of YAP1 and TAZ (Basu-Roy et al., 2015). Additionally, SOX2 has been shown to induce transcription of *Yap1* in mesenchymal stem cells and osteoprogenitors (Seo et al., 2013), whilst in developing lungs, YAP1 can induce *Sox2* expression (Mahoney et al., 2014). Therefore YAP1/TAZ is strongly associated with the stem cell state. In this manuscript we have analyzed in detail the expression of components of the Hippo pathway in the developing pituitary gland and demonstrate its activity in SOX2^+^ cells during embryonic and postnatal development.

## Materials and methods

### Animals and tissue processing

Procedures were carried out in accordance with the UK Animals (Scientific Procedures) Act 1986, subject to KCL local Ethical Review. Wild type CD1 females were mated with wild type CD1 males for the generation of embryos. *Sox2-Egfp* animals have been previously described (Ellis et al., 2004). These were maintained as heterozygotes on a CD1 background. Midday of the day of vaginal plug was considered as 0.5 days *post coitum* (dpc). Dissected embryos and postnatal tissues were fixed in 10% neutral buffered formalin (NBF) at room temperature for 36 h, then dehydrated through a graded ethanol series and processed for paraffin embedding as previously described (Gaston-Massuet et al., 2008; Sajedi et al., 2008). Samples were sectioned along the sagittal plane for embryos between 9.5dpc and 13.5dpc and frontal plane for older embryos and postnatal pituitaries, at a thickness of 7 μm for immunofluoresence and 4 μm for RNAscope mRNA *in situ* hybridization.

### Immunofluorescence

Samples were dewaxed in histoclear twice for 10 min, followed by rehydration through a descending ethanol series then washed in water. Antigen retrieval was carried out in citrate-based Declere Unmasking Solution (Cell Marque) in a steamer twice for 30 min, followed by washing in PBT. For tyramide specific amplification [TSA, for antibodies against YAP1 (Cell Signaling 4912, 1:1000), pYAP (S127) (Cell Signaling 4911S, 1:1000) and TAZ (Sigma HPA007415, 1:1000)], slides were washed in TNT buffer (0.1M Tris-HCl, pH7.5, 0.15M NaCl, 0.05% Tween-20). Slides were blocked for 1 h in TNB [0.1M Tris-HCl pH7.5, 0.15M NaCl, 0.5% Blocking Reagent (FP1020, Perkin Elmer)], and incubated with primary antibody overnight at 4°C in TNB. Following washes, species-specific biotinylated antibody was applied for 1 h at room temperature in TNB. Following washes in TNT, slides were incubated in ABC solution (Vector Laboratories, pk-6100) for 30 min in the dark then for 10 min at room temperature in TSA-Cy3 diluted in Stock Solution (Perkin Elmer, NEL760001). Slides were washed and mounted with soft-set mounting medium with DAPI (Vector Laboratories, Z1007) ready for imaging. For double immunofluorescence with GFP, the above conditions were used, with the inclusion of chicken anti-GFP primary antibody (Abcam ab13970, 1:350) and secondary goat anti-chicken Alexa Fluor 488 (Invitrogen A11039, 1:500). For antibodies against SOX2 (Abcam ab97959, 1:300) and Endomucin (Abcam ab106100, 1:500), blocking was carried out in Blocking Buffer (0.15% glycine, 2 mg/ml BSA, 0.1% Triton-X in PBS) with 10% sheep serum for minimum 1 h at room temperature. Primary antibody solution was applied overnight at 4°C, diluted in Blocking Buffer with 1% sheep serum. Slides were washed and then incubated in goat anti-rabbit biotinylated (Abcam ab6720, for α-SOX2) or goat anti-rat Alexa Fluor 633 (Life Technologies A-21094, for α-Endomucin) for 1 h at room temperature, diluted to 1:350 in Blocking Buffer with 1% sheep serum. After washing, slides were incubated in Streptavidin-488 (Life Technologies S11223) at 1:500 dilution in Blocking Buffer with 1% sheep serum for 1 h a room temperature. Slides were washed and mounted as above.

### mRNA *in situ* hybridization

Tissue sections cut at 4 μm thickness were processed for mRNA *in situ* detection using the RNAscope 2.0 Fast Red Detection Kit (Advanced Cell Diagnostics), according to manufacturer's recommendations. For 10.5dpc and 12.5dpc embryos, pre-treatment was carried out at the recommended “mild” timings and for older embryos or postnatal tissues, “standard” timings were used. RNAscope probes used: *Hesx1, Sox2, Mst1 (Stk4), Mst2 (Stk3), Lats1, Lats2, Yap1, Tead1, Tead2, Tead3, Tead4, Fat3, Fat4*, and *Dchs1* (Advanced Cell Diagnostics). To control for background, we used a negative control probe against the *Bacillus subtilis* dihydrodipicolinate reductase, *dapB* (Advanced Cell Diagnostics) (Figure S1). Sections were weakly counterstained with hematoxylin.

### Microscopy

For fluorescent images, slides were visualized on a TCS SP5 confocal system (Leica Microsystems (UK) Ltd). Images were captured using a HCX Plan-Apochromat CS 20x/0.7 dry objective and HCX Plan-Apochromat CS 63x/1.3 Glycine objective (both Leica Microsystems (UK) Ltd). The DAPI, AlexaFluor 488, 594, and 633 conjugate dyes were excited with 405, 488, 561, and 633 nm lasers respectively. Z-stack images were acquired at a total thickness of 3 μm. Images were processed for maximum intensity z-projections using Fiji (Schindelin et al., 2012). For brightfield images, slides were scanned using a NanoZoomer-XR Digital slide scanner (Hamamatsu). Panels were compiled using Adobe Photoshop to create the figures.

## Results

**Figure 1:**
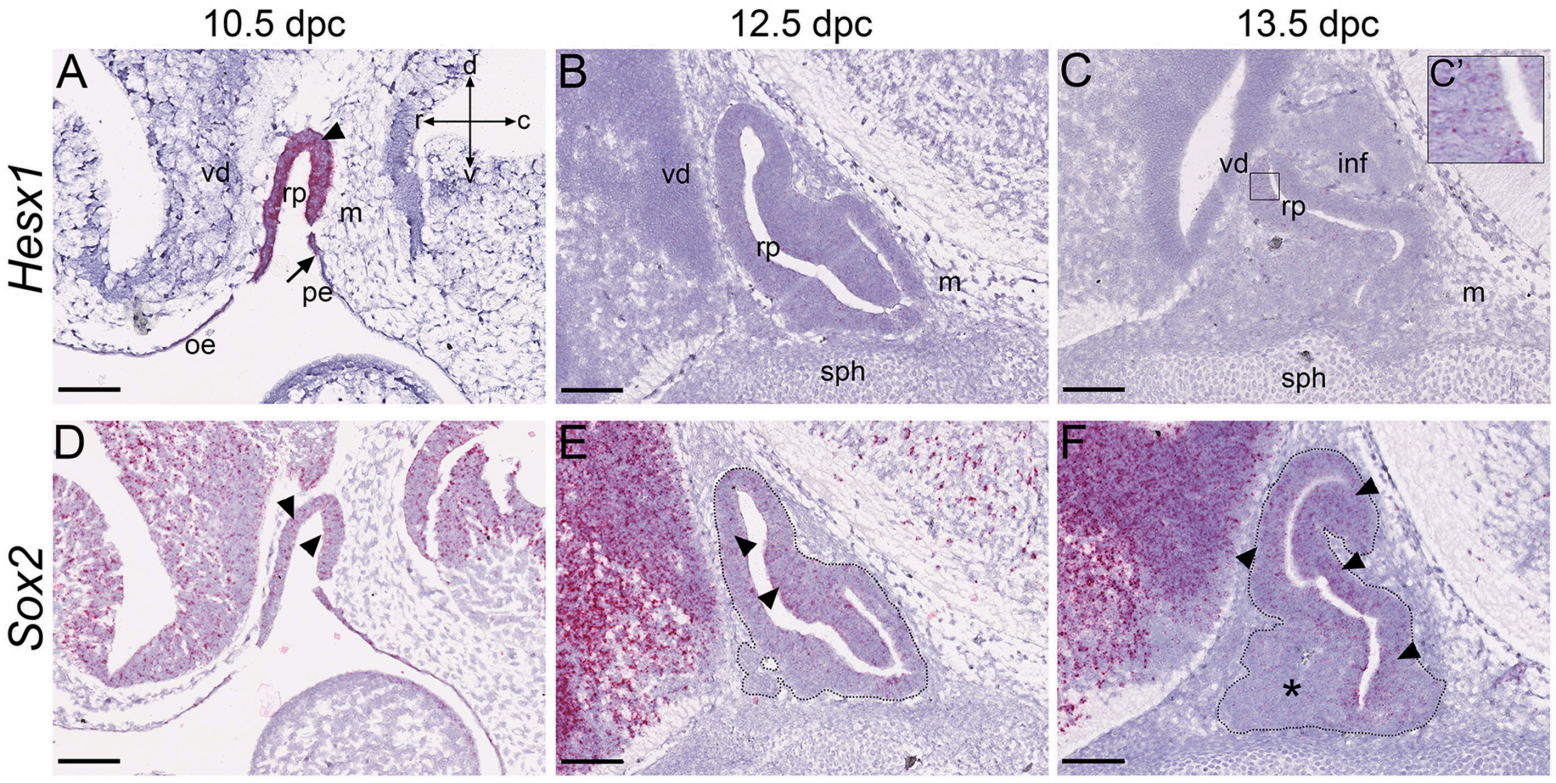
**Validation of RNAscope method in the pituitary gland**. RNAscope mRNA *in situ* hybridization using probes against *Hesx1* and *Sox2* on wild type CD1 embryos at stages between 10.5dpc and 13.5dpc. **(A–C)** At 10.5dpc *Hesx1* is expressed in the oral epithelium and Rathke's pouch (arrowhead), but excluded from the pharyngeal endoderm. The posterior limit of expression is indicated by the arrow in **(A)**. At 12.5dpc and 13.5dpc expression is reduced although still detectable (**C**′, boxed area in **C**). **(D–F)** *Sox2* is expressed in Rathke's pouch at 10.5dpc (arrowheads) as well as neural tissue, oral epithelium and pharyngeal endoderm. Expression decreases in the ventral pouch where committed cells arise (asterisk in **F**) and persists periluminal and in the dorsal RP (arrowheads in **E,F**). For clarity, the developing anterior pituitary primordium is indicated by the dotted outline in **(E,F)**. Abbreviations: rp, Rathke's pouch; vd, ventral diencephalon; m, mesenchyme; or, oral ectoderm; pe, pharyngeal endoderm; inf, infundibulum; sph, sphenoid. Sagittal sections, axes in **(A)** applicable to all panels: d, dorsal; v, ventral; r, rostral; c, caudal. Scale bars 200 μm.

To determine reliability of the RNAscope *in situ* hybridization in the pituitary we first validated this method by analysis of *Hesx1*, which has a known pattern, only expressed during development. It is expressed strongly in oral ectoderm subsequently fated to become Rathke's pouch at 8.5dpc (Cajal et al., 2012), and persists in RP epithelium until 11.5dpc with levels of expression decreasing thereafter (Thomas and Beddington, 1996). Using the RNAscope *in situ* hybridization method for sensitive mRNA detection, we confirmed strong expression of *Hesx1* in RP at 10.5dpc (Figure 1A, arrowhead), which extended rostrally in the oral epithelium. No expression was detected in the pharyngeal endoderm as previously reported (posterior limit of expression noted by arrow). Expression of *Hesx1* was barely detectable at 12.5dpc and 13.5dpc (Figures 1B,C) with presence of only sporadic transcripts (Figure 1C′ magnified boxed region in C). Additionally, we investigated expression of *Sox2*, which marks progenitors/stem cells in the pituitary, using this method. Robust expression of *Sox2* transcripts was detected in RP and the developing ventral diencephalon, with complete absence of expression in mesenchyme surrounding the pouch at all stages (Figure 1). Expression of *Sox2* is known to be down-regulated in committed lineages of the pituitary gland, which we confirmed at 13.5dpc; there is an absence of transcripts in the ventral anterior pituitary where cells are undergoing commitment (Figure 1F, asterisk). Expression persisted dorsally, specifically in the marginal epithelium surrounding the pouch where the uncommitted cells reside (arrowheads in Figure 1F).

### Expression of upstream Hippo regulators

**Figure 2:**
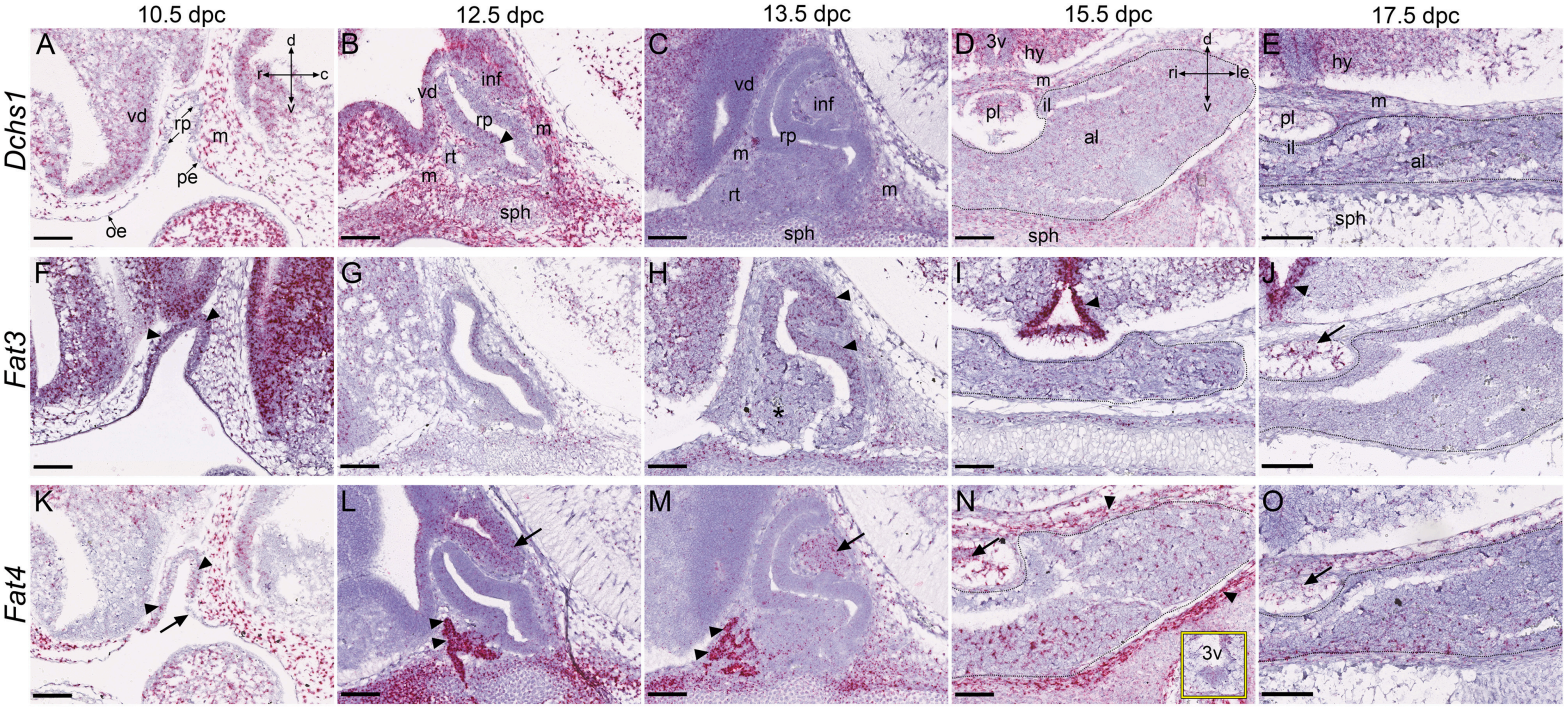
**Expression of putative upstream Hippo pathway regulators during pituitary development**. RNAscope mRNA *in situ* hybridization using probes against *Dchs1, Fat3* and *Fat4* on sections of wild type CD1 embryos between 10.5dpc and 17.5dpc. **(A–E)** *Dchs1* is expressed in mesenchyme surrounding Rathke's pouch and in neural tissue but absent from Rathke's pouch epithelium **(A)**. Expression in mesenchyme and neural tissue persists at 12.5dpc **(B)** and 13.5dpc **(C)** where transcripts are also detectable in RP (arrowhead in **B**). Expression in all pituitary lobes, the hypothalamus and surrounding mesenchyme is detectable at 15.5dpc **(D)** and 17.5dpc **(E)**. Note the reduced expression of *Dchs1* in the epithelium surrounding the third ventricle in **(D,E)**. **(F–J)** *Fat3* is strongly expressed in Rathke's pouch (arrowheads), surrounding mesenchyme, and neural tissue at 10.5dpc **(F)**. Reduced levels are detected in all tissues between 12.5dpc and 17.5dpc. At 12.5dpc there is a dorsal bias in expression in RP **(G)**, more clearly visible at 13.5dpc (arrowheads in **H**). Strong expression is detected in the epithelium surrounding the third ventricle at 15.5dpc and 17.5dpc (arrowheads in **I,J**) and in the posterior pituitary (arrow in **J**). **(K–O)** *Fat4* transcripts are detected in RP at 10.5dpc (arrowheads in **K**), in surrounding mesenchyme and neural tissue but excluded from the pharyngeal endoderm (arrow in **K**). Note the very strong expression in the rostral tip of the anterior pituitary (arrowheads in **L,M**) at 12.5pc and 13.5dpc and in the ventral diencephalon and infundibulum (arrows in **L,M**), persisting in the posterior lobe (arrows in **N,O**). Expression is high in surrounding mesenchyme (arrowheads in **N**). The box in **(N)** shows the epithelium around the third ventricle displaying low-level expression. The outlines in **(D,E,I,J,N,O)** surround anterior pituitary tissue derived from Rathke's pouch. Abbreviations: rp, Rathke's pouch; vd, ventral diencephalon; m, mesenchyme; oe, oral ectoderm; pe, pharyngeal endoderm; inf, infundibulum; sph, sphenoid; rt, rostral tip; pl, posterior lobe; al, anterior lobe; il, intermediate lobe; hy, hypothalamus; 3v, third ventricle. Sagittal sections between 10.5dpc and 13.5dpc and frontal between 15.5dpc and 17.5dpc. Axes in **(A)** applicable to (**A–C,F–H,K–M**: d, dorsal; v, ventral; r, rostral; c, caudal). Axes in **(D)** applicable to (**D–E,I–J,N–O**: d, dorsal; v, ventral; ri, right; le, left). Scale bars 200 μm.

We next sought to characterize the expression of proposed upstream regulators of the Hippo cascade, homologs of *Drosophila Ds* and *Ft*, whose protein products act as ligand-receptor pair. We analyzed expression of *Dchs1* and *Fat4* that have closest homology to *Ft* and *Ds*, as well as *Fat3*, which is detectable in developing pituitary tissue (Karine Rizzoti, personal communication). Previous studies have reported absence of expression of the remaining homologs *Fat1, Fat2*, and *Dchs2* in the pituitary gland (Diez-Roux et al., 2011). At 10.5dpc we did not observe *Dchs1* expression in RP epithelium but transcripts were detected in the caudal mesenchyme as well as in the ventral diencephalon (Figure 2A). Expression in RP was observed at 12.5dpc at low levels and persisted until at least 17.5dpc where it was detected both in the anterior and posterior pituitary and in the hypothalamus, with lowest expression in cells lining the third ventricle (Figures 2B–E). At 10.5dpc we observed robust expression of *Fat3* in the developing pouch, in ventral diencephalon and caudal mesenchyme (Figure 2F). Expression in RP persisted at lower levels until at least 17.5dpc (Figures 2G–J, arrowheads in Figures 2G,H), where strongest expression was detected in cells lining the third ventricle (arrowheads in Figures 2I,J) as well as in posterior pituitary tissue (arrow in Figure 2J). *Fat4* transcripts were present from 10.5dpc throughout RP (arrowheads in Figure 2K) and oral epithelium but excluded from the pharyngeal endoderm (arrow). Strong expression was detected in surrounding mesenchyme. At 12.5dpc very strong expression was detected at the rostral tip (arrowheads in Figure 2L) with low levels of transcripts in RP epithelium. There was also expression in the infundibulum (arrow in Figures 2L,M) and surrounding mesenchyme. *Fat4* was still expressed at 17.5dpc in sporadic cells of the anterior and posterior pituitary (arrows in Figures 2N,O) and surrounding mesenchyme (arrowheads in Figure 2N).

### Expression of pathway kinases

**Figure 3:**
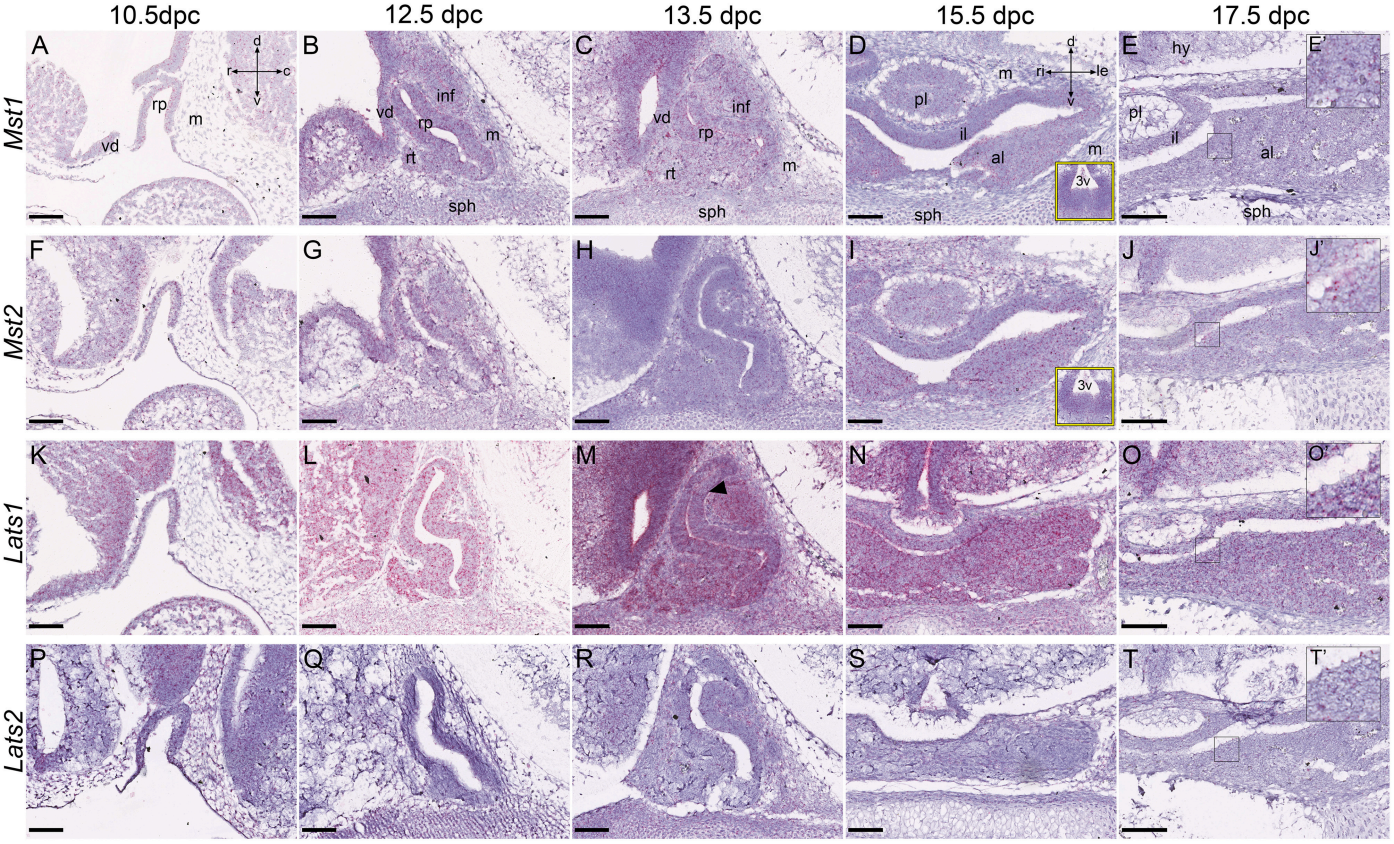
**Expression of Hippo kinase genes during embryonic development of the pituitary gland**. RNAscope mRNA *in situ* hybridization using probes against *Mst1, Mst2, Lats1*, and *Lats2* on sections of wild type CD1 embryos between 10.5dpc and 17.5dpc. **(A–J)** Transcripts of *Mst1* **(A–E)** and *Mst2* **(F–J)** are detectable in all tissues at low levels, at all stages analyzed. Yellow boxes in **(D,I)** are of the epithelium around the third ventricle. Magnifications of boxed regions in **(E,J)** at 17.5dpc show positive staining in the anterior pituitary **(E**′**,J**′**)**. **(K–O)** *Lats1* transcripts are detectable in RP and neural tissue at 10.5dpc **(K)** and persist until 17.5dpc (**L–O** and **O**′ magnification of boxed region in **O**). Note the ventral bias in expression in RP at 13.5dpc (arrowhead in **M**). Lower levels of expression are detected in surrounding mesenchyme. **(P–T)** Very low levels of *Lats2* transcripts are detected in all tissues at all stages analyzed (note positive expression in **T'**, magnification of boxed region in **T** at 17.5dpc). Abbreviations: rp, Rathke's pouch; vd, ventral diencephalon; m, mesenchyme; inf, infundibulum; sph, sphenoid; rt, rostral tip; pl, posterior lobe; al, anterior lobe; il, intermediate lobe; hy, hypothalamus; 3v, third ventricle. Sagittal sections between 10.5dpc-13.5dpc and frontal between 15.5dpc and 17.5dpc. Axes in **(A)** applicable to (**A–C**,**F–H**,**K–M**,**P–R**: d, dorsal; v, ventral; r, rostral; c, caudal). Axes in **(D)** applicable to (**D,E,I,J,N,O,S,T**: d, dorsal; v, ventral; ri, right; le, left). Scale bars 200 μm.

In multiple tissues, MST1 and MST2 have redundant functions. At all stages analyzed, expression of both *Mst1* and *Mst2* was observed throughout the developing pituitary between 10.5dpc and 17.5dpc (Figures 3A–J). We detected salt and pepper expression in Rathke's pouch, the infundibulum and their subsequent derivatives. Both genes were also expressed in neural structures from 10.5dpc with few sporadic cells displaying transcripts in surrounding mesenchyme at 10.5dpc and 12.5dpc.

We observed strong *Lats1* expression at all stages throughout the oral epithelial-derived and neural tissues (Figures 3K–O). From 13.5dpc we observed a ventral bias in the developing pituitary, with the dorsal epithelium surrounding the lumen (future intermediate lobe) displaying lower expression (arrowhead in Figure 3M). As seen for *Mst1*/*Mst2*, expression in the mesenchyme was only detected in occasional single cells at 10.5dpc but was abundant from 12.5dpc. Expression of *Lats2* was very low at all stages analyzed, across the developing pituitary and surrounding tissues (Figures 3P–T), but detectable (Figure 3T′, magnified boxed area in Figure 3T).

### Expression of Hippo pathway effectors

**Figure 4:**
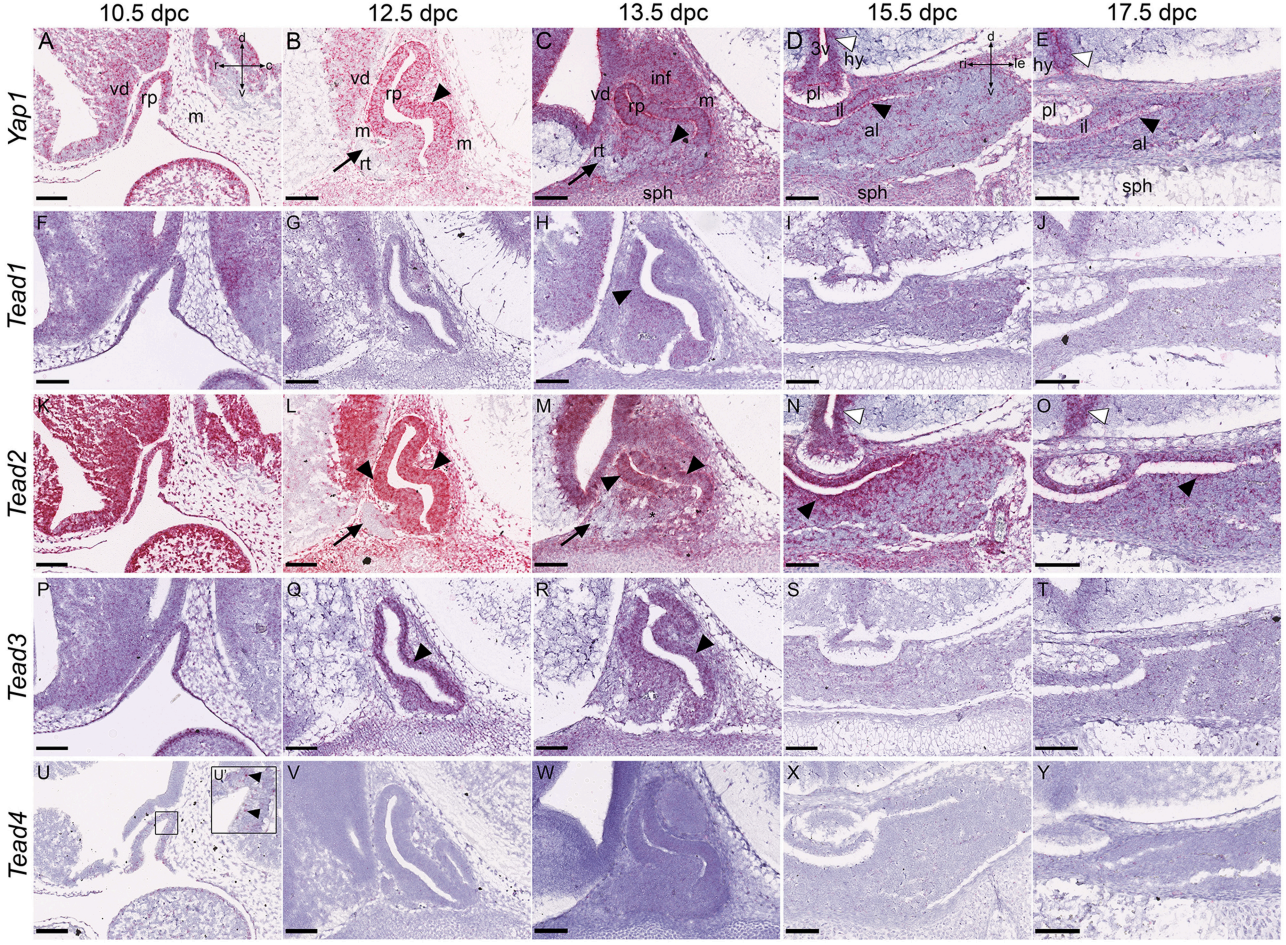
**Expression of the Hippo pathway effectors during embryonic development of the pituitary gland**. RNAscope mRNA *in situ* hybridization using probes against *Yap1, Tead1, Tead2, Tead3*, and *Tead4* on sections of wild type CD1 embryos between 10.5dpc and 17.5dpc. **(A–E)** *Yap1* transcripts are detected in neural tissue, mesenchyme and Rathke's pouch epithelium between 10.5dpc and 13.5dpc (**A–C**, arrowheads indicating RP expression). Note the dorsal expression bias at 12.5dpc and 13.5dpc and absence of transcripts in the rostral tip (arrows in **B,C**). Transcripts persist in all tissues at 15.5dpc and 17.5dpc especially in the periluminal region (black arrowhead in **D**) and epithelium surrounding the third ventricle (white arrowheads in **D,E**). **(F–Y)** Expression of *Tead1, Tead2, Tead3*, and *Tead4* encoding TEAD transcription factors. *Tead1* and *Tead3* transcripts are detectable in all tissues at low levels **(F–J,P–T)**, higher in RP (arrowheads in **F,H,P–R**). *Tead2* is highly expressed in all tissues at 10.5dpc **(K)**, and from 12.5dpc becomes restricted to the ventral diencephalon in neural tissue **(L,M)** and to the epithelium surrounding the third ventricle (white arrowheads in **N,O**). *Tead2* is strongly expressed in the periluminal epithelium of the anterior pituitary primordium (black arrowheads **L–O**) but excluded from the rostral tip (arrows **L,M**). *Tead4* transcripts are barely detectable **(U–Y)**. Abbreviations: rp, Rathke's pouch; vd, ventral diencephalon; m, mesenchyme; inf, infundibulum; sph, sphenoid; rt, rostral tip; pl, posterior lobe; al, anterior lobe; il, intermediate lobe; hy, hypothalamus; 3v, third ventricle. Sagittal sections between 10.5dpc and 13.5dpc and frontal between 15.5dpc and 17.5dpc. Axes in **(A)** applicable to (**A–C,F–H,K–M,P–R,U–W**: d, dorsal; v, ventral; r, rostral; c, caudal). Axes in **(D)** applicable to (**D,E,I,J,N,O,S,T,X,Y**: d, dorsal; v, ventral; ri, right; le, left). Scale bars 200 μm.

When YAP1 and TAZ are not phosphorylated by LATS kinases they can associate with TEAD transcription factors in the nucleus to promote expression of target genes. We sought to determine the expression patterns of *Yap1* and *Tead1-Tead4* in the developing pituitary. We were not able to determine the expression of *Taz* mRNA using this method. *Yap1* showed robust expression in Rathke's pouch and surrounding tissues at 10.5dpc (Figure 4A). By 12.5dpc there was strong expression in the dorsal aspect of the pouch in the epithelium (Figure 4B arrowhead), reduced expression in the expanding ventral portion and no expression in the rostral tip (arrow). This pattern was maintained at 13.5dpc and new tissue in the ventral region that is undergoing commitment showed low expression (arrowhead in Figure 4C). At 15.5 and 17.5dpc, *Yap1* transcripts remained strong in the intermediate lobe, marginal zone of the anterior lobe and in scattered groups of cells throughout the anterior lobe (Figures 4D,E, black arrowheads). *Yap1* was expressed in neural tissue and surrounding mesenchyme at all stages, maintained until 17.5dpc when it was expressed in the posterior lobe, the cell layer surrounding the third ventricle (white arrowheads in Figures 4D,E) and in mesenchyme-derived connective tissue surrounding the gland. From the four *Tead* genes, *Tead2* expression was the strongest (Figures 4K–O). This was reminiscent of the expression pattern of *Yap1*: strong expression in RP and all surrounding tissues at 10.5dpc (Figure 4K), strong expression in RP epithelium at 12.5dpc and 13.5dpc (Figures 4L,M, arrowheads) but no expression in the rostral tip (Figures 4L,M, arrows) and reduced expression in the ventral pituitary primordium at 13.5dpc (Figure 4M, asterisk). *Tead2* expression remained very high at 15.5dpc in the marginal zone (Figure 4N, black arrowhead) and intermediate lobe and in scattered groups of cells throughout the anterior lobe. Levels of expression were reduced but present at 17.5dpc (Figure 4O, black arrowhead). The posterior lobe also expressed high levels of *Tead2*, as did cells surrounding the third ventricle (Figures 4N,O, white arrowheads). *Tead1* and *Tead3* were expressed at low levels at all stages examined (Figures 4F-J, P-T) and *Tead4* was not expressed (Figures 4V-Y) except for low levels of transcript in RP at 10.5dpc only (Figures 4U,U′ arrowheads).

### Localization of YAP1 and TAZ proteins

**Figure 5:**
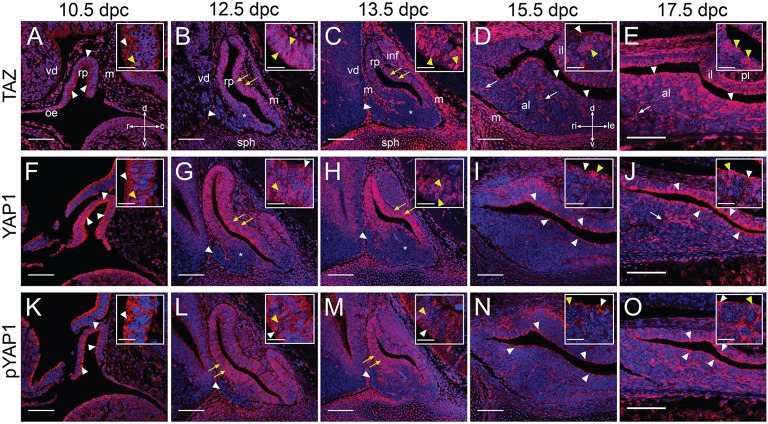
**Expression of YAP1 and TAZ proteins during embryonic development**. Immunofluorescence using specific antibodies against total TAZ protein, total YAP1 protein and phosphorylated YAP1 (S127) in red. Nuclei are counterstained with DAPI (blue). **(A–J)** Localization of effectors TAZ **(A–E)** and YAP1 **(F–J)** using antibodies recognizing total protein. Note the nuclear localization in periluminal cells of Rathke's pouch (yellow arrowheads in **B,C,G,H**, white arrowheads in **D,E,I,J**). No YAP1/TAZ proteins are detected in the rostral tip (arrowheads in **B,C,G,H**) and there is a reduction in expression in ventral regions (asterisks in **B,C,G,H**). Note expression in structures resembling capillaries from 15.5dpc (arrows in **D,E,J**). **(K–O)** Immunofluorescence to detect the phosphorylated form of YAP1 at S127. Protein detection indicates Hippo kinase cascade activity at all stages, primarily in periluminal RP epithelium (white arrowheads in **K,N,O**). Note the ventral bias of protein localization at 12.5dpc and 13.5dpc (yellow arrows in **L,M**) and complete absence of protein from the rostral tip (white arrowheads in **L,M**). Boxed inserts show higher magnifications of the epithelium. Examples of cytoplasmic localization are noted by white arrows and examples of nuclear localization by yellow arrows. Abbreviations: rp, Rathke's pouch; vd, ventral diencephalon; m, mesenchyme; oe, oral ectoderm; inf, infundibulum; sph, sphenoid; rt, rostral tip; pl, posterior lobe; al, anterior lobe; il, intermediate lobe. Sagittal sections between 10.5dpc and 13.5dpc and frontal between 15.5dpc and 17.5dpc. Axes in **(A)** applicable to (**A–C,F–H,K–M**: d, dorsal; v, ventral; r, rostral; c, caudal). Axes in **(D)** applicable to (**D,E,I,J,N,O**: d, dorsal; v, ventral; ri, right; le, left). Scale bars 100 μm and 20 μm in boxed inserts.

In order to infer activity of the Hippo kinase cascade, we investigated the localization of effector proteins TAZ, total YAP1, as well as the inactive phosphorylated form of YAP1 (S127). TAZ and YAP1 had similar localization at 10.5dpc; they appeared nucleo-cytoplasmic with a bias for the apical cytoplasm of RP epithelium (Figures 5A,F, arrowheads). Inactive YAP1, marked by pYAP1 was strongly cytoplasmic and also displayed an apical bias (Figure 5K, arrowheads). All three antibodies marked cells in the mesenchyme and neural tissue. At 12.5dpc and 13.5dpc YAP1 and TAZ both localized mostly in nuclei of cells in RP epithelium, with stronger expression in the dorsal RP epithelium at 12.5dpc, which persisted at 13.5dpc for YAP1 (yellow arrowheads in Figures 5B,G,H). In the ventral portion of the epithelium there was nuclear localization in a thin cell layer surrounding the cleft. Little expression was observed in more ventral regions (asterisk in Figures 5B,C,G,H), and no expression in the rostral tip (arrowheads in Figures 5B,C,G,H). Phosphorylated YAP1 was cytoplasmic in cells both in the dorsal and ventral regions at both stages but completely absent from the rostral tip (arrowheads in Figures 5L,M). Expression was stronger in the ventral epithelium than the dorsal (yellow arrowheads in Figures 5L,M), the reverse of the observed pattern for total YAP1 and TAZ. At 15.5dpc and 17.5dpc YAP1 and TAZ were nucleo-cytoplasmic in the marginal zone epithelium surrounding the cleft on both sides, in the intermediate and anterior lobes (arrowheads in Figures 5D,E,I,J). TAZ protein was detected in a broader domain surrounding the epithelium than YAP1 (Figure 5E). They were both present in cells scattered around the anterior pituitary and in structures resembling blood vessels (arrows in Figures 5D,E,J). Inactive phosho-YAP1 was present in the cytoplasm of cells in the marginal zone epithelium (arrowheads in Figures 5N,O) and in many cells throughout the anterior and intermediate lobes at both stages. The posterior lobe stained with all three antibodies, nucleo-cytoplasmic for TAZ and cytoplasmic for YAP1 and phospho-YAP1.

**Figure 6:**
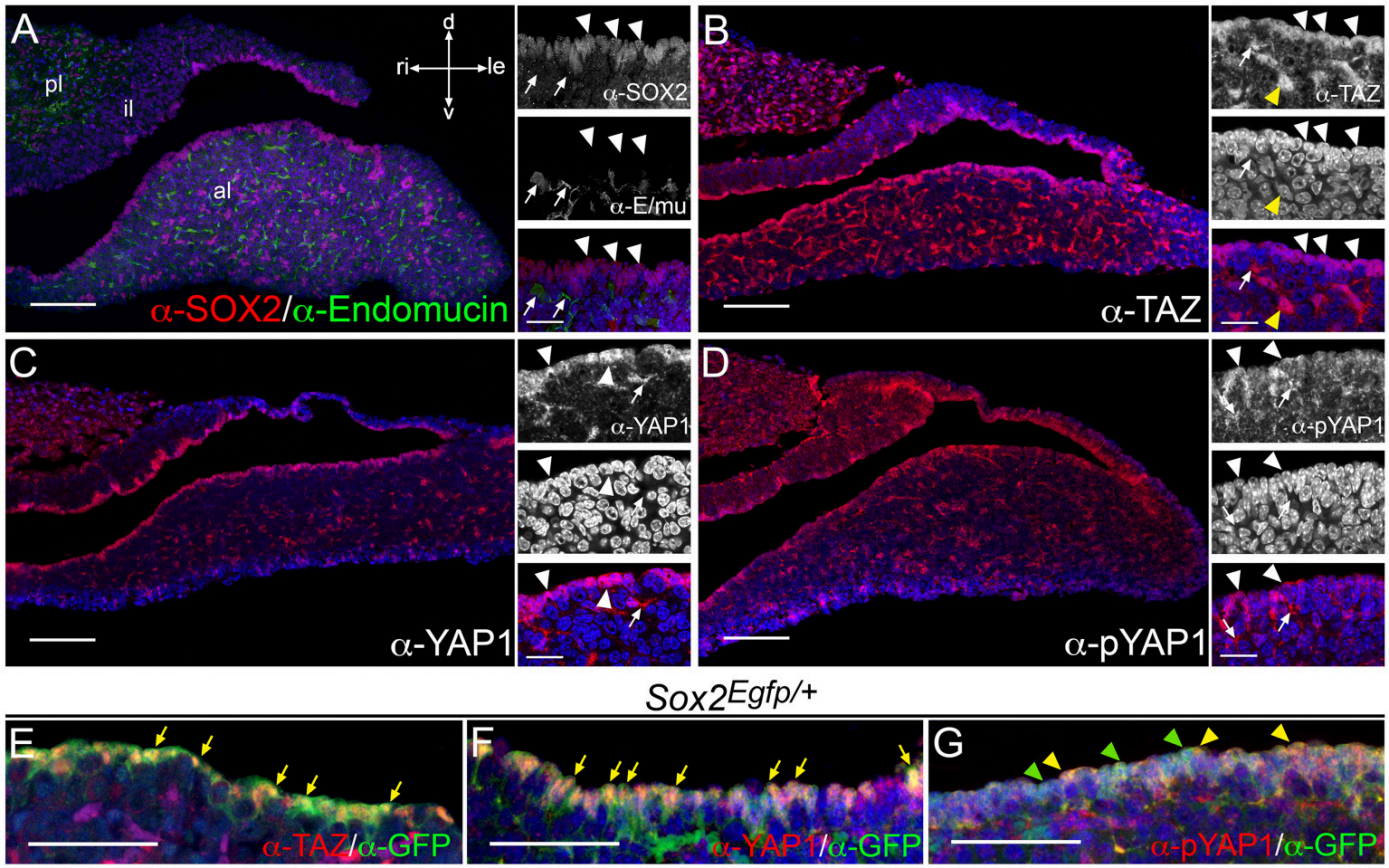
**Expression of YAP1 and TAZ proteins in postnatal pituitaries**. Immunofluorescence using specific antibodies on frontal sections of dissected postnatal pituitaries at P21. **(A)** Immunofluorescence using antibodies against the pituitary stem cell marker SOX2 (red, arrowheads) showing positive staining in the periluminal epithelium and in small groups of cells throughout the anterior lobe parenchyma. Antibodies against endomucin mark epithelial cells surrounding blood vessels (green, arrows). **(B–D)** Immunofluorescence staining against total TAZ protein **(B)**, total YAP1 protein **(C)** and phosphorylated YAP1 (S127) protein **(D)** in red. All three are detected in periluminal cells (white arrowheads) as well as structures resembling blood vessels (white arrows). **(E–G)** Double immunofluorescence staining on pituitary sections from *Sox2*^*Egfp*∕+^ animals using antibodies against GFP protein in green detecting EGFP and marking SOX2^+^ cells together with antibodies against TAZ protein **(E)**, total YAP1 protein **(F)** and phosphorylated YAP1 (S127) protein **(G)** in red. Arrows in **(E,F)** indicate examples of cells with nuclear TAZ or YAP1 protein. Yellow arrowheads in G note examples of GFP^+^ cells with strong nucleocytoplasmic pYAP1 protein localization and green arrowheads indicate GFP^+^ cells in lower pYAP1 levels in the nucleus. Nuclei are counterstained with DAPI (blue). Abbreviations: pl, posterior lobe; al, anterior lobe; il, intermediate lobe. Axes in **(A)** applicable to all panels: d, dorsal; v, ventral; ri, right; le, left. Scale bars 100 μm in **(A–D)**, 20 μm in inserts and 50 μm in **(E–G)**.

We next investigated the localization of TAZ, YAP1, and phospho-YAP1 in sections of postnatal pituitary glands at P21, a time following the peak postnatal proliferative stage, when the gland is still expanding and undergoing major hormonal profile changes at weaning. We found that YAP1 and TAZ were primarily nuclear in epithelial cells lining the pituitary cleft (Figures 6B,C), which express the stem cell marker SOX2 (Figure 6A, arrowheads). Nucleo-cytoplasmic staining was also seen in cells associated with blood vessels (arrows in inserts in Figures 6B,C). The pattern of blood vessels was revealed by staining using antibodies against endomucin (Figure 6A, arrows). Nucleo-cytoplasmic staining for YAP1 and TAZ was also seen in scattered cells throughout the anterior lobe, where higher proportions were positive for TAZ (yellow arrowheads in Figure 6B, inserts). Inactive phosphorylated YAP1 was localized in the cytoplasm of cells in the marginal zone epithelium, stronger in some regions (arrowheads in Figure 6D indicating stronger staining). There was diffuse expression in the anterior pituitary and cytoplasmic staining in cells lining blood vessels (arrows in Figure 6D). In order to determine localization of TAZ, YAP1 and pYAP1 specifically in SOX2^+^ cells we carried out double immunofluorescence staining on sections at P21 from *Sox2*^*Egfp*∕+^ animals, using antibodies against GFP to mark SOX2^+^ cells (Figures 6E–G). We find nuclear localisation of both TAZ and YAP1 in cells positive for GFP (arrows in Figures 6E,F). Levels of pYAP1 in the epithelium do not correlate with GFP positivity, with some GFP^+^ cells showing stronger staining for pYAP1 protein (yellow arrowheads in Figure 6G) and lower levels in others (green arrowheads in Figure 6G).

## Discussion

Coordinating proliferation, differentiation and cell death is critical for normal development of tissues and for maintaining the balance of cells during long-term homeostasis. The Hippo kinase cascade has been shown to mediate these processes through the inhibition of proliferation and promotion of differentiation and cell death. In this manuscript we reveal that the Hippo signaling cascade is active during all stages of embryonic pituitary development assessed and continues to act in the postnatal organ.

The genes *Mst1* and *Mst2* encoding the core Hippo kinases, are both expressed throughout the developing pituitary and *Lats1* is expressed at high levels during development. Since we barely detected expression of *Lats2*, we hypothesize the main kinase upstream of YAP1/TAZ in the gland is likely to be *Lats1*. From the four *Tead* genes that encode the pathway transcription factors, *Tead2* is the highest expressed making it likely to act as the main regulator of downstream target gene transcription. Interestingly, expression of *Yap1* is very similar to *Tead2*, which are both strongest expressed in the regions rich in stem/progenitor cells. Expression of both is completely absent from the rostral tip of the pituitary at 12.5dpc and 13.5dpc, as is expression of YAP1 and TAZ proteins and of phosphorylated YAP1, despite positive expression of the kinases *Mst1, Mst2*, and *Lats1* in this tissue. This suggests that the Hippo cascade is not regulating the rostral tip, but this region highly expresses *Fat4*, which can act as a receptor upstream of the Hippo cascade. Taken together, we hypothesize that *Fat4* is not acting upstream of Hippo in the rostral tip during development. Expression of *Dchs1* that complements the receptor-ligand interaction is strong in mesenchyme surrounding Rathke's pouch, likely acting in concert with FAT4 at the rostral tip. Both genes are expressed at low levels in the anterior pituitary and a possible role upstream of the Hippo cascade cannot be excluded. From this gene family, *Fat3* is also a candidate to encode a protocadherin upstream of the pathway; its expression in RP resembles that of *Yap1* and *Tead2*, as well as *Sox2*. These demonstrate a dorsal bias in expression, at the region of the future intermediate lobe and stem cell-containing region of the anterior lobe. Expression in the stem cell-rich periluminal zone persists in the anterior pituitary at later stages.

We observe strong nuclear localization of YAP1 and TAZ throughout the stem cell-rich regions of developing Rathke's pouch and the postnatal anterior pituitary and reveal that phosphorylation of YAP1 at S127 occurs, suggesting kinase activity within the SOX2^+^ stem cell pool. The S127 residue is in one of the five LATS phosphorylation consensus motifs and results in YAP1 regulation by the Hippo pathway through 14-3-3 binding and cytoplasmic retention (Zhao et al., 2007). Activity of the kinase cascade on the stem/progenitor pool in the pituitary gland may function to regulate stem cell numbers or behavior. Additionally, we observe localization of all three proteins in blood vessels in the gland, which will need to be taken into account during any interpretation of future functional data for this pathway in the pituitary. At 12.5dpc the ventral epithelium of Rathke's pouch has stronger pYAP1 expression, suggesting that the Hippo cascade may have higher activity in the ventral, more committed aspect, corroborated by stronger nuclear expression of total YAP1 and TAZ in the dorsal epithelium. Several inputs can influence Hippo cascade activity, such as mechanotransduction, polarity and G-protein-coupled receptor signaling. More recently, negative regulation of the cascade by the transcription factor SOX2 was shown, which is expressed by many stem cell types, including pituitary stem cells throughout development and postnatal stages. SOX2 has been shown to antagonize the function of the Hippo cassette in two ways: by directly regulating *Yap1* transcription (Seo et al., 2013) as well as by antagonizing NF2 and WWC1, homologs of Merlin and Kibra respectively, that positively regulate MST1/2 thus resulting in reduced phosphorylation of YAP1/TAZ (Basu-Roy et al., 2015). This places SOX2 as a likely candidate upstream of the Hippo cascade in the pituitary gland, and its potential role to maintain the stem/progenitor cell state through this pathway can be addressed in future.

Our results suggest there is appropriate expression of Hippo pathway components to support a possible functional role in the pituitary gland. A previous study reporting over-proliferation of uncommitted pituitary tissue in the absence of LATS1 kinase (St John et al., 1999) supports that this pathway may be acting to regulate homeostasis in the pituitary. It remains to be determined if the function of LATS1 in the gland is mediated through YAP1/TAZ. In summary, our results highlight that Hippo signaling is active in the pituitary gland, both during development and at postnatal stages and reveal the expression patterns of its major components. Our studies suggest a possible role for this pathway in the regulation of the uncommitted stem/progenitor cell pool, with its function remaining to be elucidated.

## Author contributions

CA, EL conceived the study; EL, JR conducted the experiments with support from AP; EL, JR, and CA analyzed the results. PF provided intellectual contribution. CA, EL wrote the manuscript. All authors reviewed and approved the final manuscript.

### Conflict of interest statement

The authors declare that the research was conducted in the absence of any commercial or financial relationships that could be construed as a potential conflict of interest.

## Supplementary material

Figure S1 **Negative controls for RNAscope method in the pituitary gland**. RNAscope mRNA *in situ* hybridization using probes against *dapB*, encoding bacterial dihydrodipicolinate reductase, on wild type CD1 embryos at stages between 10.5dpc and 13.5dpc. **(A–E)** Represenative examples of negative controls used in rounds of RNAscope to determine background levels of expression. For each stage analyzed, rare to no red dots were observed. Inserts are magnifications of boxed regions. Axes in **(A)** applicable to **(A–C)**; axes in **(D)** applicable to **(D–E)**. Scale bars 250 μm in **(A–D)** and 100 μm in **(E)**.
