# Supplementary figures and images for: Expression Analysis of the Hippo Cascade Indicates a Role in Pituitary Stem Cell Development
Source: Front Physiol. 2016 Mar 31;7:114. doi: 10.3389/fphys.2016.00114 (PMC4814506; doi:10.3389/fphys.2016.00114)

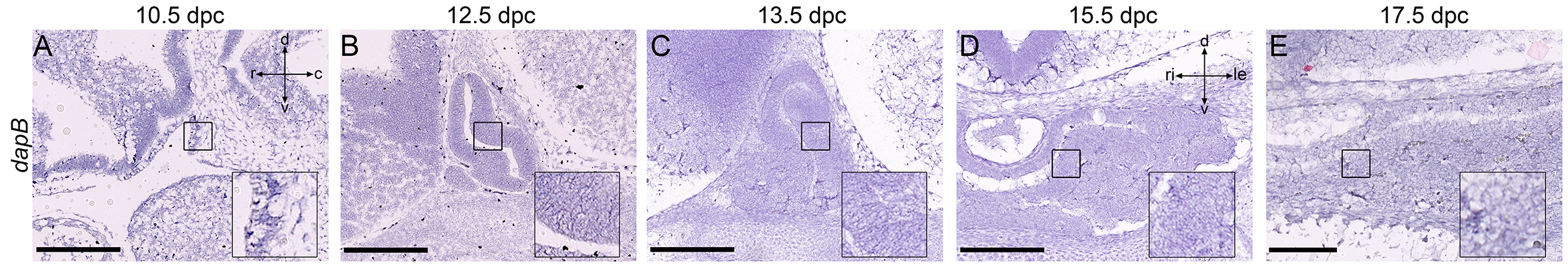

Supplement: Figure S1 — Negative controls for RNAscope method in the pituitary gland. RNAscope mRNA in situ hybridization using probes against dapB, encoding bacterial dihydrodipicolinate reductase, on wild type CD1 embryos at stages between 10.5dpc and 13.5dpc. (A–E) Represenative examples of negative controls used in rounds of RNAscope to determine background levels of expression. For each stage analyzed, rare to no red dots were observed. Inserts are magnifications of boxed regions. Axes in (A) applicable to (A–C); axes in (D) applicable to (D–E). Scale bars 250 μm in (A–D) and 100 μm in (E). [file Image1.JPEG]
